# The Submucosal Microbiome Correlates with Peri-implantitis Severity

**DOI:** 10.1177/00220345251352809

**Published:** 2025-07-28

**Authors:** A.A. Joshi, S.P. Szafrański, M. Steglich, I. Yang, W. Behrens, P. Schaefer-Dreyer, J. Grischke, S. Häussler, M. Stiesch

**Affiliations:** 1 Department of Prosthetic Dentistry and Biomedical Materials Science, Hannover Medical School, Hannover, Germany; 2 Lower Saxony Centre for Biomedical Engineering, Implant Research and Development (NIFE), Hannover, Germany; 3 Cluster of Excellence RESIST (EXC 2155), Hannover Medical School, Hannover, Germany; 4 Department of Molecular Bacteriology, Helmholtz Centre for Infection Research, Braunschweig, Germany; 5 Institute for Molecular Bacteriology, Twincore, Centre for Clinical and Experimental Infection Research, Hannover, Germany; 6 Department of Clinical Microbiology, Copenhagen University Hospital–Rigshospitalet, Copenhagen, Denmark

**Keywords:** biomarkers, dental implant(s), genomics, microbiology, peri-implant infection(s), plaque/plaque biofilms

## Abstract

Peri-implantitis is an increasingly prevalent chronic inflammatory disease of the peri-implant tissue. A key etiologic factor for peri-implantitis is the submucosal biofilm, which may further drive clinical severity and accelerate disease progression. The present cross-sectional study aimed to characterize the compositional (full-length 16S rRNA gene amplicon sequencing) and functional patterns (metatranscriptomics) in the microbiome as an indicator of peri-implantitis severity. For this purpose, submucosal biofilm samples were collected from 49 peri-implantitis–diagnosed implants in 34 patients. Notable microbial signatures were associated with increased probing depth (PD), a measure for disease severity. Multivariate linear regression analysis, adjusted for patient variability, showed that the genera *Capnocytophaga* and *Gemella* were negatively correlated with increased peri-implantitis severity whereas *Pseudoramibacter* was positively correlated with it. PICRUSt2-based metabolic pathway prediction revealed the following to be negatively correlated with PD: central carbon metabolism, nitrate reduction, sulfate assimilation, glycolysis, the tricarboxylic acid cycle, and heme biosynthesis. In contrast, cobalamin and tetrahydrofolate biosynthesis showed positive correlations. Metatranscriptomic analysis uncovered additional enzyme functions correlated with PD, which were related to galactose metabolism and proteolysis. Our data allowed the proposal of an extended microbial dysbiosis index for peri-implantitis severity. The quantitative index, integrating significant microbial and functional features, revealed high correlation with PD. In conclusion, our results showed that the level of peri-implantitis severity is associated with distinct and significant changes in microbial composition as well as microbial functions. The severity-specific microbiome signatures identified in our study will advance microbiome-based diagnostics and disease stratification, paving the way for targeted clinical interventions for peri-implantitis.

## Introduction

Peri-implantitis is a biofilm-associated pathologic condition affecting the tissue around dental implants. It is characterized by inflammation of the peri-implant mucosa and progressive loss of supporting bone (Berglundh et al. 2018). Depending on the extent of inflammation and tissue damage, peri-implantitis can present at varying levels of severity, which correlate with clinical indicators such as probing depth (PD), bleeding on probing, and/or suppuration (Schwarz et al. 2018).

A key etiologic factor for peri-implantitis is submucosal microbial dysbiosis (Daubert and Weinstein 2019; Belibasakis and Manoil 2021). However, until today, the specific microbial taxa and their functional profiles in dysbiosis that correlate with disease severity have not been fully elucidated. Yet, a clear ecologic and mechanistic understanding of the relationship between dysbiotic changes in the submucosal microbiome and peri-implantitis severity is crucial for the further development of microbiome-based diagnostics. Moreover, understanding this relationship may support effective disease stratification, enabling development of targeted preventive and treatment strategies, particularly for severe forms of peri-implantitis that often present with unpredictable clinical outcomes (Lagervall and Jansson 2013).

In recent years, advances in next-generation sequencing technologies have improved our understanding of the submucosal microbiome around dental implants (Kumar et al. 2012; Zheng et al. 2015; Sanz-Martin et al. 2017; Ghensi et al. 2020; Polymeri et al. 2021). Yet, most of these omics-based studies have focused on differences in the microbiome composition between health and peri-implantitis or between periodontitis and peri-implantitis, not on peri-implantitis severity. For periodontitis, subgingival microbiome profiles have been described for various disease stages (Tonetti et al. 2018; Lafaurie et al. 2022; Iniesta et al. 2023), but comparable evidence for peri-implantitis is either limited or indirectly derived (Maruyama et al. 2014; Kröger et al. 2018; Polymeri et al. 2021; Yu et al. 2024). Although studies have provided initial clues about associations between submucosal microbial dysbiosis and the severity of peri-implant disease, a comprehensive functional characterization is still lacking. However, a functional characterization of the submucosal microbiome is essential for elucidating the underlying molecular mechanisms and is therefore more informative than the composition alone, given the polymicrobial complex nature of peri-implantitis biofilms (Komatsu et al. 2020).

Hence, the aim of the present study was to characterize the peri-implantitis–associated submucosal microbiome with high-throughput full-length 16S rRNA gene amplicon sequencing (full-16S) and metatranscriptomics (RNAseq) to identify the microbial taxa and functional bacterial and enzymatic activities associated with different levels of PD, as an indicator of disease severity. Furthermore, we aimed to develop an easy-to-interpret quantitative index integrating microbial taxa and functions to stratify peri-implantitis severity.

## Material and Methods

### Cohort Characteristics and Clinical Examination

The present cross-sectional study is a part of the interdisciplinary consortium Safety Integrated and Infection Reactive Implants, which aims to characterize the etiopathogenesis of peri-implant diseases and develop early detection and prevention strategies. The study was conducted at the Department of Prosthetic Dentistry and Biomedical Materials Science, Hannover Medical School, in accordance with the STROBE guidelines (Strengthening the Reporting of Observational Studies in Epidemiology). The study protocol was approved by the ethics committee of Hannover Medical School, Germany (No. 9477).

The Appendix provides a detailed description of patient selection criteria (Appendix Table 1), clinical examination, biofilm sample collection, DNA-RNA co-isolation, sequencing, and bioinformatic and statistical methods. The sequencing data for this study have been deposited in the NCBI SRA as BioProject PRJNA1192962.

## Results

The study population comprised 34 patients (17 male, 17 female) with a mean ± SD age of 73 ± 9 y and 49 implants diagnosed with peri-implantitis. The population- and site-specific characteristics of the study cohort are presented in Appendix Table 2. Implants were in function for 9.8 ± 6 y. The mean PD of the implants was 7.1 ± 2.3 mm (range, 5 to 11 mm). All implants showed bleeding on probing, and suppuration was observed in 41%.

### Diversity and Variability of Full-16S Microbiome Profiles in Peri-implantitis Samples

**Figure 1.**
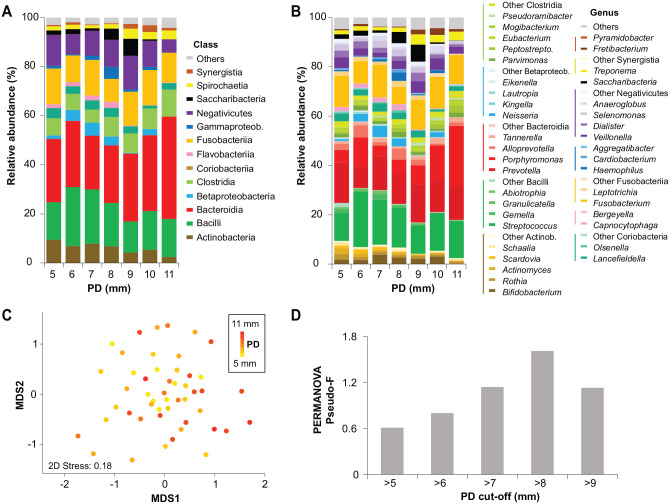
Microbial community distribution and variability across peri-implantitis samples. (**A**, **B**) Class- and genus-level relative abundances averaged across peri-implantitis samples based on PD. (**C**) nMDS plot based on the Bray-Curtis dissimilarity matrix of square root–transformed species-level relative abundances, with samples colored by PD values. (**D**) Results of the PERMANOVA, including pseudo-*F* statistics, illustrate group differences at various PD cutoffs. nMDS, non-metric multi-dimensional scaling; PD, probing depth; PERMANOVA, permutational multivariate analysis of variance.

Full-16S yielded 715,134 sequences (mean, 14,594 sequences per sample) after filtering and preprocessing of reads. Following removal of typical contaminants (Appendix Methods), we identified 599 species-level taxa belonging to 13 phyla, 20 classes, 33 orders, 53 families, and 93 genera. High taxonomic diversity was captured with the included number of samples (Appendix Fig. 1), with no change in alpha diversity across PD levels. Figure 1A illustrates that Bacteroidia was the most abundant class across all PD levels, followed by Bacilli, Negativicutes, and Fusobacteriia. Figure 1B displays the distribution of genera within these classes. The distinct variation in microbial composition at the species level was seen along the MDS1 axis as visualized by a non-metric multi-dimensional scaling (nMDS) ordination plot (Fig. 1C). We tested different PD thresholds and selected the cutoff based on permutational multivariate analysis of variance pseudo-*F* values. The analysis yielded the highest pseudo-*F* value of 1.6, when samples were divided into 2 groups at a PD cutoff of 8 mm. The nonsignificant PermDisp result (*P* = 0.76) indicated homogeneity of within-group sample variability. On the basis of this microbiome-driven criterion, we categorized the samples into 2 groups: group 1, comprising samples with PD ≤8 mm; group 2, comprising samples with PD >8 mm. This allowed us to analyze PD as a categorical and continuous variable.

### Correlation of Full-16S Microbial Taxa with Peri-implantitis Severity

**Figure 2.**
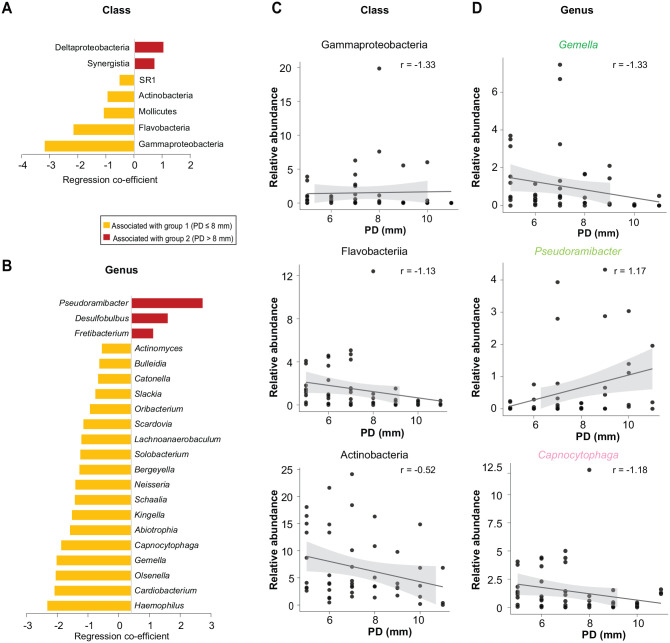
Correlation of microbial taxa with PD groups and distinct PD levels. (**A**, **B**) Class- and genus-level taxa significantly associated with the 2 PD groups (categorical). (**C**, **D**) Scatter plots show classes and genera with significant correlations to peri-implantitis severity, calculated with “PD” as a continuous fixed variable. All regression correlations were calculated with “patient” as a random effect in MaAsLin2 analysis, with a default adjusted *P* value significance threshold <0.25. PD, probing depth.

Microbiome differences between groups 1 and 2 were analyzed by MaAsLin2 and DESeq2. MaAsLin2 showed that classes Gammaproteobacteria, Flavobacteriia, Mollicutes, Actinobacteria, and SR1 were significantly associated with group 1, while Deltaproteobacteria and Synergistia were significantly associated with group 2 (Fig. 2A). At the genus level, several genera were significantly associated with group 1 (Fig. 2B). Most significant of those were *Gemella*, *Schaalia*, *Haemophilus*, *Olsenella*, and *Capnocytophaga*, while *Pseudoramibacter*, *Desulfobulbus*, and *Fretibacterium* were positively correlated with an increase in PD. Differentially abundant species were identified at a log_2_ fold change >2 (adjusted *P* < 0.05) only in the dataset unadjusted for patients (Appendix Fig. 2).

Next, we analyzed PD as a continuous variable, as displayed in Figure 2C and D, and identified microbial taxa significantly associated (adjusted *P* < 0.25) with PD using a linear regression approach in MaAsLin2 adjusted for patients as a random effect. The classes Gammaproteobacteria, Flavobacteriia, and Actinobacteria were negatively correlated with an increase in PD. At the genus level, *Gemella* and *Capnocytophaga* were negatively correlated with PD. Additional subgenus analysis for *Capnocytophaga* showed that the major species within it consistently exhibited a negative relationship with PD (Appendix Fig. 3). At the sequence level, the median amplicon sequence variance richness decreased with increasing PD severity and was significantly lower when the 2 PD groups were compared (*t* test, *P* = 0.0034). *Pseudoramibacter* was positively correlated with an increase in PD and key peri-implant pathogens (e.g., *Porphyromonas*, *Tanerella*, *Fretibacterium*; Appendix Fig. 4).

The comparison of different approaches (PD continuous vs PD groups) and methods (DESeq2 and MaAsLin2) showed the highest correlation (*r* = 0.93) between MaAsLin2 models and for DESeq2 and MaAsLin2 models based on PD groups (*r* = 0.7) (Appendix Fig. 2D-F). Among the other clinical parameters, the combination of plaque index, gingival index, and Periotron could best explain the microbial composition patterns (BIO-ENV, ρ = 0.183, *P* = 0.059, 10,000 permutations).

### Prediction of Functional Potential of Biofilms in Peri-implantitis

**Figure 3.**
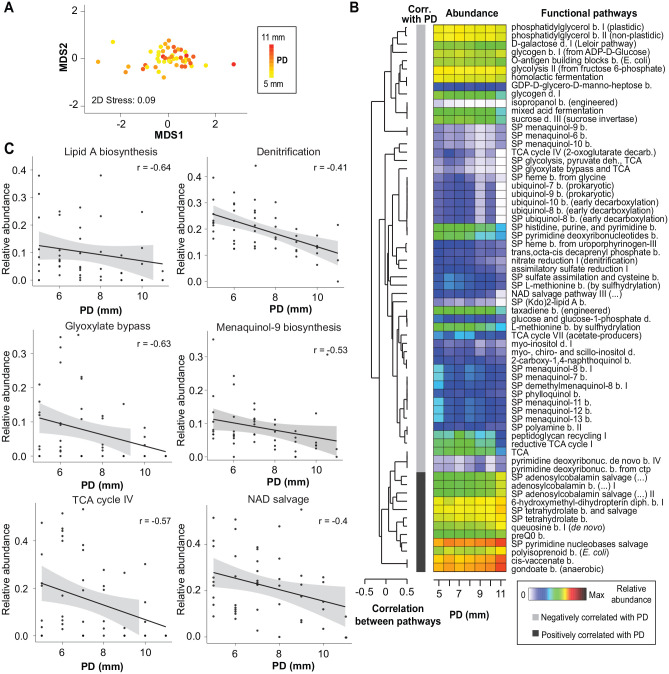
Correlation of predicted MetaCyc functional pathways at different levels of PD. (**A**) nMDS plot based on Bray-Curtis dissimilarity matrix of square root–transformed pathway-level relative abundances of PICRUSt2 predictions, with samples colored by PD values. (**B**) Heat map shows the relative abundances of predicted MetaCyc functional pathways (MaAsLin2 default adjusted *P* < 0.25) averaged for PD across samples. Pathways are clustered according to Spearman rank correlation. b, biosynthesis; d, degradation; SP, superpathway. (**C**) Scatter plots of pathways with the highest significant correlations to changes in PD. All regression correlations were calculated with “patient” as a random effect variable. nMDS, non-metric multi-dimensional scaling; PD, probing depth.

Prediction of functional potential within biofilms based on the 16S rRNA marker gene revealed 261 MetaCyc pathways with relative abundances >0.1% in at least 1 peri-implantitis sample. The nMDS ordination plot illustrates a substantial overlap of PDs across MDS1, with a slight tendency of clustering among higher PD samples (Fig. 3A). A heat map displays the relative abundances of significant predicted MetaCyc functional pathways (MaAsLin2 default adjusted *P* < 0.25) (Fig. 3B). Among the pathways negatively correlated with PD, several are involved in core metabolic functions: lipopolysaccharide biosynthesis (superpathway of Kdo_2_–lipid A biosynthesis), central carbon metabolism (superpathway of glyoxylate bypass and tricarboxylic acid; superpathway of glycolysis, pyruvate dehydrogenase, and tricarboxylic acid) and cofactor biosynthesis (menaquinol, ubiquinol, and heme biosynthesis). Pathways linked to nitrogen and sulfur metabolism, including nitrate reduction I and assimilation sulfate reduction I, exhibited negative correlations with PD. In contrast, positively correlated pathways were predominantly associated with stress response mechanisms, and cell wall and capsule biosynthesis (e.g., polyisoprenoid biosynthesis, fatty acid elongation pathway). Cofactor biosynthesis, including superpathways for cobalamin and tetrahydrofolate biosynthesis, was positively correlated with increased PD. From the stated pathways, the scatterplots of 6 pathways with the highest regression correlation values (−0.4 to −0.6) are shown in Figure 3C. Appendix Table 3 demonstrates predicted enzyme commission (EC)–level activities that were correlated with PD. Strikingly, when EC-level functional prediction was analyzed for samples matched for metatranscriptomics (*n* = 27), only 6 predicted ECs demonstrated a significant association with PD, with none showing a positive correlation.

### Metatranscriptomic Functional Activities at Different Levels of Peri-implantitis Severity

In contrast to PICRUSt2-based predicted enzyme functions, the RNAseq revealed a high number of true enzymatic activities that were significantly associated with PD (adjusted *P* < 0.25), as shown in the Table. Of 24 EC numbers, 21 were negatively correlated, while 3 ECs belonging to the serine and aspartic endopeptidase enzyme class were positively correlated with PD. The negatively correlated ECs belonged to the KEGG metabolic pathways associated with carbohydrate, amino acid, amino sugar, nucleotide sugar, pyruvate, and butyrate metabolism.

**Table.** Metatranscriptomic ECs Significantly Correlated with PD (Adjusted *P* < 0.05) across the Samples Included for RNAseq (*n* = 27).

EC No.	Enzyme Name	Regression Coefficient	SE	*P* Value	Adjusted *P* Value
5.4.99.9	UDP-galactopyranose mutase	−0.545	0.139	0.001	0.176
4.1.2.40	Tagatose-bisphosphate aldolase	−1.085	0.290	0.001	0.176
3.4.21.102	C-terminal processing peptidase	0.226	0.061	0.001	0.176
3.4.22.70	Sortase A	−0.564	0.161	0.002	0.185
3.4.21.53	Endopeptidase La	0.148	0.045	0.003	0.185
3.4.11.2	Membrane alanyl aminopeptidase	−0.698	0.214	0.003	0.185
2.7.7.9	UTP-glucose-1-phosphate uridylyltransferase	−0.367	0.111	0.003	0.185
2.7.1.204	D-galactose PTS permease	−1.166	0.368	0.004	0.185
3.4.23.36	Signal peptidase II	0.146	0.046	0.004	0.185
1.1.1.38	Malate dehydrogenase	−0.727	0.233	0.005	0.185
2.7.7.27	Glucose-1-phosphate adenylyltransferase	−0.514	0.165	0.005	0.185
5.3.1.26	Galactose-6-phosphate isomerase	−1.053	0.338	0.005	0.185
2.2.1.6	Acetolactate synthase	−0.536	0.177	0.006	0.213
2.7.1.144	Tagatose-6-phosphate kinase	−0.886	0.311	0.009	0.232
2.8.3.18	Succinyl-CoA:acetate CoA-transferase	−0.655	0.229	0.009	0.232
2.3.3.13	2-Isopropylmalate synthase	−0.517	0.182	0.009	0.232
2.7.3.9	Phosphoenolpyruvate-protein phosphotransferase	−0.554	0.199	0.010	0.232
1.12.99.6	Hydrogenase	−0.735	0.257	0.010	0.232
2.3.3.1	Citrate (Si)-synthase	−0.476	0.167	0.011	0.232
6.3.4.13	Phosphoribosylamine-glycine ligase	−0.125	0.044	0.011	0.232
2.7.1.191	Protein-Npi-phosphohistidine-D-mannose phosphotransferase	−0.729	0.261	0.011	0.232
3.2.1.20	Alpha-glucosidase	−0.802	0.293	0.011	0.232
5.4.2.2	Phosphoglucomutase (alpha-D-glucose-1,6-bisphosphate-dependent)	−0.234	0.086	0.011	0.232
5.3.1.1	Triose-phosphate isomerase	−0.212	0.078	0.011	0.232

Positive regression coefficient refers to the positive correlation of ECs with PD and vice versa.

EC, enzyme commission; PD, probing depth.

### Extended Microbial Dysbiosis Index for Peri-implantitis Severity

**Figure 4.**
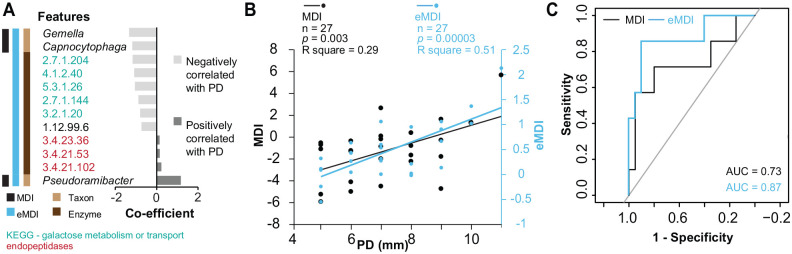
Correlation of MDI and eMDI with different PD levels. (**A**) Top genus-level taxa and enzyme commission features included in eMDI for peri-implantitis severity and their regression correlation values in relation to PD. (**B**) Scatter plot of genus-level MDI and eMDI shows significant correlation with PD. Linear regression lines fitted separately for MDI and eMDI. (**C**) Area under the receiver operating characteristic curve evaluating the performance of the genus-level MDI and eMDI to distinguish peri-implantitis samples into group 1 (PD ≤8 mm) and group 2 (PD >8 mm). AUC, area under the curve; eMDI, extended microbial dysbiosis index; MDI, microbial dysbiosis index; PD, probing depth.

To quantify the microbial dysbiosis related to severity and to develop a clinically relevant index with a reduced number of features, we first calculated the microbial dysbiosis index (MDI) using 3 significant PD-associated genus-level taxa: *Capnocytophaga*, *Gemella*, and *Pseudoramibacter*. A significant positive correlation was observed between the MDI and PD in the full-16S dataset (*n* = 49, *P* = 0.0002) and in the smaller dataset matching RNAseq samples (*n* = 27, *P* = 0.003; Appendix Fig. 5A). To integrate the RNAseq EC features to the MDI, full-16S and RNAseq EC features were standardized across 27 samples. The resulting MDI according to scaled data ranged from −5.9 (least dysbiotic) to +5.6 (most dysbiotic), with 85% below zero for group 1 and 57% above zero for group 2 (*R*^2^ = 0.29, *P* = 0.003). Incorporating RNAseq EC data through sequential feature addition enhanced the performance of the MDI (Appendix Fig. 5B). The resulting extended MDI (eMDI) included the aforementioned 3 genera and the following 9 enzymes: 5 ECs associated with galactose metabolism, 1 hydrogenase, and 3 endopeptidases (Fig. 4A). Although some variability was observed in a few samples, the relative abundances of the included ECs showed a consistent increase or decrease across PD levels (Appendix Fig. 6) and were not clustered with the genera included in the eMDI based on Spearman correlations (Appendix Fig. 7). The eMDI achieved the strongest correlation with PD (*R*^2^ = 0.51, *P* = 0.00003; Fig. 4B) and showed higher accuracy (area under the curve, 0.87) than only the genus-based MDI (area under the curve, 0.73) in distinguishing between the PD groups (Fig. 4C).

## Discussion

The present study comprised 49 submucosal taxonomic and functional profiles of implants diagnosed with peri-implantitis. We observed clear differences in the microbiome with changes in PD and identified significant associations of microbial taxa, potential functional pathways, and transcriptome-level specific enzymatic activities with peri-implantitis severity. A microbiome-based index (eMDI) was introduced for the first time, which is specific to peri-implantitis severity and integrates taxa and their enzymatic activities.

Our study integrated 2 high-throughput approaches, full-16S and metatranscriptomics, to characterize the severity of peri-implantitis. Sequencing the full-length 16S rRNA gene (~1,500 base pairs) increased reliability and precision of taxonomic assignments (e.g., identification of species for a greater proportion of sequences) as compared with short-read sequencing platforms (Johnson et al. 2019). Furthermore, it is recognized that community composition alone does not adequately reflect the intricate biological processes occurring within peri-implant biofilms representing a range of molecular elements (Solbiati and Frias-Lopez 2018; Belibasakis and Manoil 2021). We employed metatranscriptomics for 27 peri-implantitis samples to identify RNA-level traits revealing the true microbial activities associated with severity of peri-implantitis, thus addressing the need for functional biomarker candidates (Belibasakis and Manoil 2021) while contributing to a deeper understanding of oral dysbiosis (Solbiati and Frias-Lopez 2018). To date, only 17 peri-implantitis metatranscriptomes have been published, originating from 2 studies (Shiba et al. 2016; Ganesan et al. 2022), both of which compared peri-implantitis with either periodontitis or health, without specifically addressing disease severity. Our data provide a foundation for scaling up these comparisons, which is critically needed (Belibasakis and Manoil 2021). Metatranscriptomics has uncovered host-microbiome interactions (Ganesan et al. 2022), presenting an intriguing aspect for further studies across varying PDs.

Our full-16S approach revealed that although peri-implantitis biofilms represent highly complex and diverse microbial communities, core and severity-associated taxa can be identified. *Prevotella*, *Porphyromonas*, and *Fusobacteria* species representing well-established periodontopathogens dominated biofilms independently of PD, which aligns with previous findings on peri-implantitis microbiomes (Sanz-Martin et al. 2017; Ghensi et al. 2020). *Streptococcus* species, which are generally associated with peri-implant health, were abundant in peri-implantitis. Additionally, our study identified significant levels of disease-associated taxa, such as *Dialister*, *Fretibacterium*, *Selenomonas*, and *Megasphaera*, whose physiology remains poorly understood (Koyanagi et al. 2013; Ghensi et al. 2020; Yu et al. 2024). Interestingly, we reported significant associations of *Capnocytophaga gingivalis*, *Capnocytophaga sputigena*, and *Capnocytophaga leadbetteri* with shallower pockets. *Capnocytophaga* species have been linked to periodontal health and disease (Savitt and Socransky 1984; Holdeman et al. 1985). A recent full-16S study (Yu et al. 2024) reported higher abundances of the aforementioned *Capnocytophaga* species in peri-implant pockets of 5 to 7 mm. These findings, with our results, suggest that these species may be associated with the early stages of peri-implantitis. *Pseudoramibacter* was positively associated with pocket depths and showed a potential link to peri-implantitis severity across cohorts (Kröger et al. 2018; see Appendix Discussion). We hypothesize that fastidious *Pseudoramibacter* serves as an indicator of prolonged dysbiosis, rather than being directly involved in the destructive process.

Another highlight of our study was the functional analyses employing 16S-based predictions. Abundances of certain metabolic pathways and their contributing taxa were associated with PD. For instance, the nitrate reduction pathway and *Neisseria*, a genus known for its strong nitrate-reducing capacity (Rosier et al. 2024), were both negatively associated with severity. Deeper peri-implant pockets, however, were associated with increased predictive abundances of cobalamin and tetrahydrofolate salvage, which are known to be activated as an alternative to de novo synthesis of these cofactors in pathogenic bacteria (Kuboniwa et al. 2017). These findings suggest that disease severity is driven not only by the changes in microbial composition but also by metabolic adaptations that enable microbial establishment in shallower pockets and persistence in the inflammatory and anaerobic environment of deeper pockets. Further details of our key findings and their ecologic implications are discussed in the Appendix.

Our study is the first to utilize metatranscriptomics to assess the expressed enzymatic activities (ECs) associated with peri-implantitis severity. Although full-16S–based functional prediction revealed broad, pathway-level significant associations with PD, it lacked the resolution of metatranscriptomics and did not capture a high number of specific enzymatic functions that were uniquely detected through metatranscriptomics. The strongest negative correlations were observed for ECs that belong to galactose metabolism, while 2 serine endopeptidases and an aspartic endopeptidase showed positive correlation with deeper peri-implant pockets. Previous periodontal and peri-implant metatranscriptomic studies have positively linked the expression of various peptidases to disease, whereas multiple carbohydrate-related pathways, including galactose metabolism and phosphotransferase systems, are shown to have an inverse association (Duran-Pinedo and Frias-Lopez 2015; Szafranski et al. 2015; Yost 2015; Nowicki et al. 2018). Utilization of galactose (Takahashi 2015) present on the surfaces of host proteins appeared to be an important adaptation in periodontopathogens to the early peri-implantitis environment, while the production of proteolytic activities potentially capable of degrading host-derived glycoproteins and tissue components could serve as a reliable marker for disease severity (Eley and Cox 2003). Further analysis of peptidases showed that *Fusobacterium*, *Porphyromonas*, and *Prevotella* species were the main producer taxa, consistent with early phenotypic findings for oral isolates (Beighton et al. 1997). We identified these enzymes as promising markers as they represent collectively similar activities performed by several distinct species (Eley and Cox 2003). A better understanding of how bacteria utilize these enzymes could offer valuable insights for developing protease inhibitor–based strategies, similar to those developed against other endopeptidases (Ksiazek et al. 2015). Furthermore, fluorogenic protease substrates targeting specific endopeptidase activities are widely available and represent a promising approach for diagnostic development. Thus, our findings provide a basis for further studies to elucidate the role of functional activities related to microbial dysbiosis in peri-implantitis, particularly associated with shifts from carbohydrate to protein catabolism in disease progression.

In this context, the eMDI for peri-implantitis severity proposed by us showed high accuracy for severity-based peri-implantitis stratification and may be relevant for clinical evaluation of peri-implantitis progression. Previous efforts (Kröger et al. 2018; Shi et al. 2022; Feng et al. 2024) have successfully quantified the microbial dysbiosis in peri-implant diseases and underscored the association between the level of dysbiosis and PD. However, there still remains a need for an objective microbiome-based index that uses a smaller number of high-sensitivity and high-accuracy biomarkers, making it suitable for chair-side diagnostics. This gap could be addressed with the index presented in this study. The key advantage of eMDI lies in integrating taxonomic and taxon-independent enzymatic features as biomarkers. The panel of limited candidate markers—including 3 genera (*Capnocytophaga*, *Gemella*, and *Pseudoramibacter*) and 9 enzymes (5 ECs associated with galactose metabolism, 1 hydrogenase, and 3 endopeptidases)—captures bacterial succession associated with clinical changes, as well as proteolytic activity that may be causally linked to those changes.

This study reveals important microbiome differences linked to peri-implantitis severity. However, limitations should be considered. The cross-sectional design limits causal inference and prognostic implications, underscoring the need for longitudinal validation. Moreover, a relatively small number of severe peri-implantitis samples (PD >8 mm, *n* = 14) may have limited the identification of positive associations with an increase in PD. Additionally, the advanced age and presence of smokers (*n* = 2) are known to significantly influence host immune responses, microbiome composition, and the inflammatory milieu (Tsigarida et al. 2015; Moradi et al. 2025), although in our cohort the adjusted analyses retained our key findings (Appendix Methods). Although the achieved sequencing coverage was high, low-abundance amplicon sequence variances and low-activity ECs may still be underrepresented due to sequencing depth limitations. The default significance threshold in MaAsLin2 is higher (*q* ≤ 0.25) than the conventional threshold typically used in clinical research. Yet, this is designed to account for the increased multiple-testing burden in the multivariable approach of MaAsLin2 (Mallick et al. 2021), which is considered acceptable in exploratory microbiome studies with high-dimensional data (Jiang et al. 2017). This approach enhances sensitivity for detecting potential associations but compromises specificity, which may affect the robustness and translational relevance of the findings. Future studies with larger independent cohorts will be necessary to confirm definitive clinical significance.

In conclusion, this study is the first to integrate DNA-based taxonomic and RNA-based functional analyses of peri-implantitis microbiomes in relation to disease severity. Our findings demonstrated significant associations of microbiome composition and functional activities with peri-implantitis disease severity. Additionally, we proposed a microbiome-based index (eMDI) that strongly correlates with different disease severities. Our high-resolution characterization of the peri-implantitis microbiome is an important step toward disease stratification and the development of targeted therapeutic strategies based on the level of peri-implantitis severity.

## Author Contributions

A.A. Joshi, contributed to data acquisition, analysis, and interpretation, drafted and critically revised the manuscript; S.P. Szafrański, contributed to data acquisition, analysis, and interpretation, critically revised the manuscript; M. Steglich, I. Yang, W. Behrens, contributed to data analysis, critically revised the manuscript; P. Schaefer-Dreyer, contributed to data acquisition and analysis, critically revised the manuscript; J. Grischke, contributed to data acquisition, critically revised the manuscript; S. Häussler, M. Stiesch, contributed to data conception and design, critically revised the manuscript. All authors gave final approval and agree to be accountable for all aspects of the work.

## Supplemental Material

sj-docx-1-jdr-10.1177_00220345251352809 – Supplemental material for The Submucosal Microbiome Correlates with Peri-implantitis Severity Supplemental material, sj-docx-1-jdr-10.1177_00220345251352809 for The Submucosal Microbiome Correlates with Peri-implantitis Severity by A.A. Joshi, S.P. Szafrański, M. Steglich, I. Yang, W. Behrens, P. Schaefer-Dreyer, J. Grischke, S. Häussler and M. Stiesch in Journal of Dental Research

sj-docx-2-jdr-10.1177_00220345251352809 – Supplemental material for The Submucosal Microbiome Correlates with Peri-implantitis Severity Supplemental material, sj-docx-2-jdr-10.1177_00220345251352809 for The Submucosal Microbiome Correlates with Peri-implantitis Severity by A.A. Joshi, S.P. Szafrański, M. Steglich, I. Yang, W. Behrens, P. Schaefer-Dreyer, J. Grischke, S. Häussler and M. Stiesch in Journal of Dental Research
