# Supplementary material for: The Submucosal Microbiome Correlates with Peri-implantitis Severity
Source: J Dent Res. 2025 Jul 28;105(3):313–22. doi: 10.1177/00220345251352809 (PMC12861548; doi:10.1177/00220345251352809)
Supplement: sj-docx-1-jdr-10.1177_00220345251352809 – Supplemental material for The Submucosal Microbiome Correlates with Peri-implantitis Severity [file sj-docx-1-jdr-10.1177_00220345251352809.docx]

**Appendix**

**The Submucosal Microbiome correlates with Peri-implantitis Severity**

**Author list -** Amruta A. Joshi^1,2^, Szymon P. Szafrański^1,2,3^, Matthias Steglich^1,2^, Ines Yang^1,2^, Wiebke Behrens^1,2^, Paula Schaefer-Dreyer^1^, Jasmin Grischke^1^, Susanne Häussler^3,4,5,6^, and Meike Stiesch^1,2,3^

^1^Department of Prosthetic Dentistry and Biomedical Materials Science, Hannover Medical School, Hannover, Germany

²Lower Saxony Centre for Biomedical Engineering, Implant Research and Development (NIFE), Hannover, Germany

^3^Cluster of Excellence RESIST (EXC 2155), Hannover Medical School, Hannover, Germany

^4^Department of Molecular Bacteriology, Helmholtz Centre for Infection Research, Braunschweig, Germany

^5^Institute for Molecular Bacteriology, Twincore, Centre for Clinical and Experimental Infection Research, Hannover, Germany.

^6^Department of Clinical Microbiology, Copenhagen University Hospital - Rigshospitalet, Copenhagen, Denmark

**Corresponding author -** Meike Stiesch, Department of Prosthetic Dentistry and Biomedical Materials Science, Hannover Medical School, Carl-Neuberg-Str.1, 30625 Hannover, Germany. Stiesch.Meike@mh-hannover.de

**Appendix Methods**

***Cohort characteristics and clinical examination***

Systemically healthy patients attending a regular implant maintenance program (Armitage and Xenoudi 2016; Grischke et al. 2019) with at least one implant diagnosed with peri-implantitis according to the AAP-EFP classification (Berglundh et al. 2018), were enrolled at the Department of Prosthetic Dentistry and Biomedical Materials Science, Hannover Medical School. Accordingly, the predefined criteria for peri-implantitis included presence of bleeding on probing (BoP) and/or suppuration, increased peri-implant probing depth (PD) and radiographic signs of bone loss as compared to previous examinations, or PD ≥6 mm and bone loss ≥3 mm in absence of previous examinations. The detailed inclusion and exclusion criteria for study participants is reported in Appendix Table 1. All participants provided informed consent and completed a health questionnaire detailing general health, smoking habits and treatment history. PD was measured at six points per implant using a standardized periodontal probe (CP15 Probe, Hu-Friedy Mfg. Co. LLC, Chicago, USA), with the deepest value being documented to categorize the severity of peri-implantitis. Additional clinical parameters including BoP, suppuration, gingival index (GI) and plaque index (PI) were recorded to document the condition of peri-implant tissues.

***Submucosal biofilm sampling and co-isolation of DNA-RNA***

Submucosal biofilm samples were collected from 49 implants in 34 patients using a sequential approach to maximize biomass recovery. First, peri-implant sulcular fluid and biofilm were collected by inserting paper points (ISO 35/2.0, VDW GmbH, Munich, Germany) into the peri-implant pockets around each implant for 30 seconds. This was followed by insertion of a Gracey curette (SG1/2RXE2, Hu-Friedy Mfg. Co. LLC, Chicago, USA) to collect additional submucosal plaque biomass from the same area. The collected material was then transferred onto a fresh sterile paper point. Finally, all paper points from each implant were pooled in an Eppendorf tube containing 1 ml RNAprotect (Qiagen, Hilden, Germany) and stored at -80 °C until further processing. Sample size was determined based on appropriate considerations for sequencing depth and biological variability as well as based on previous experience (Szafranski et al. 2015) and existing literature on similar studies (Shiba et al. 2016, Ganesan et al. 2022). The study included 27 metatranscriptomes, more than double the sample sizes of the aforementioned studies, and in the range for oral biofilms (Solbiati and Frias-Lopez 2018). On average we generated 34 million quality-controlled (QC) reads per sample. While aiming for a minimum sequencing depth of 1 million QC non-human, non-ribosomal reads per sample, we obtained 1.6 million to 15 million of these reads, with an average of 6.2 ± 3.8 million reads (s.d.) per sample. Taxon accumulation and rarefaction curves based on full-16S data at both the species and genus levels reached a plateau across all samples (Appendix Figure 1), indicating that the sequencing depth and sample size were sufficient to capture the microbial diversity present in peri-implantitis biofilms.

DNA and RNA isolation from the biofilm samples was performed by modifying the previously published RNA isolation protocol (Grischke et al. 2021). The paper points were thawed and shredded with sterile scissors. The fragmented paper points were incubated in lysis buffer containing 10 mM Tris, 1 mM EDTA, pH 8.0, 2.5 mg/ml lysozyme and 50 U/ml mutanolysin at 25 °C for 1.5 h on a shaking incubator at 350 r.p.m. A total 700 μl of fresh buffer RLT (Qiagen) containing 1% (v/v) β-mercaptoethanol was added and vortexed for 10 sec. Samples (including the fragmented paper points) were placed on a QIAshredder Mini Spin column (Qiagen) and centrifuged for 1 min at 11,000 r.p.m. The flow-through containing the bacterial cells was mixed with 150 mg acid-washed and autoclaved glass beads (diameter 106 μm). Samples were vortexed 10 times for 30 sec at full speed with at least 1-min intervals on ice in between vortexing. The samples were then centrifuged for 1 min at maximal speed. Total RNA was isolated from the supernatants using the RNeasy Mini Kit (Qiagen). DNA was removed by on-column digestion and by DNase digestion in the eluate using the RNeasy cleanup procedure. Once the eluate for RNA extraction had been separated, DNA isolation was performed by placing the RNAeasy minispin columns in new tubes and incubated with 30 µl of 8 mM NaOH at 55 °C for 10 min. Eluate was collected by centrifugation and 3.03 µl 0.1M HEPES was added to it and stored at -80 °C until further use.

***Full length 16S rRNA gene amplicons and mRNA sequencing***

The full-length 16S rRNA gene amplicons extracted were amplified from each sample using the universal primer set 27F (AGRGTTYGATYMTGGCTCAG) and 1492R (RGYTACCTTGTTACGACTT). The amplification process was carried out with the KAPA PCR mix, involving 23–27 cycles of denaturation at 95 °C for 30 seconds, annealing at 55 °C for 30 seconds, and extension at 72 °C for 90 seconds, followed by a final synthesis step at 72 °C for 10 minutes. After amplification, quality control was performed using the Qubit dsDNA and BR Assay Kit (Invitrogen, Waltham, Massachusetts, USA) along with the Qubit 2.0 fluorometer (Thermo Fisher Scientific, Waltham, Massachusetts, USA). The amplified PCR products were purified using the AMPure bead purification protocol. Samples with a DNA concentration of 5 ng or more proceeded to the next stage of PacBio sequencing (PacBio Biosciences Inc, California, USA). SMRTbell libraries were prepared from the amplified DNA following the manufacturer's guidelines. Circular consensus sequence (CCS) reads were generated from the raw sequencing data using the standard software tools provided by PacBio.

For mRNA sequencing, ribosomal RNA from eukaryotic cytoplasmic and mitochondrial sources, as well as bacterial ribosomal RNA, was depleted using removal probes and magnetic beads with the Ribo-Zero Kit Epidemiology (Illumina, San Diego, CA, USA). The quality and quantity of total RNA, enriched mRNA, and synthesized cDNA were evaluated using the 2100 Bioanalyzer instrument along with the RNA 6000 Pico Kit and the High Sensitivity DNA Kit (Agilent Technologies Inc, Santa Clara, USA). Transcriptomic libraries were prepared using the ScriptSeq v2 RNA-Seq Kit (Illumina). Sequencing was performed in single-end mode, producing reads of 50 or 68 base pairs, on an Illumina HiSeq 2500 sequencer with the TruSeq SBS Kit v3—HS (Illumina).

***Full-16S rRNA gene amplicons and RNA sequencing analysis***

CCS sequences from PacBio sequencing were analyzed using an in-house pipeline (Dieckow et al. 2024). Sequences were identified to species level taxa by analysis of BLAST results (BLAST+ v2.5.0) obtained from a modified bacteria-only version of the SILVA SSU Ref_NR 99 database version 132 (Quast et al. 2013) enriched with Human Oral Microbiome Database (HOMD)_16S_rRNA RefSeq Version 15.1 sequences (Chen et al. 2010), and the All-Species Living Tree Project (LTP) database version LTPs132_SSU (Yarza et al. 2008), supplemented with unnamed and phylotype sequences from HOMD 16S_rRNA RefSeq. BLAST settings for species identification within the 16S pipeline were “-evalue 1E-100 -perc_identity 0.95”. Hits with identity above 0.97 were compared based on an id score calculated by multiplying the BLAST identity value with the relative hit length excluding gaps. The relative hit length was obtained as the inverse of the query or hit sequence lengths, whichever was shorter, multiplied by the BLAST match length on the corresponding sequence. Reads that could not be reliably assigned to one species were clustered into 97% identity OTUs using UPARSE (Edgar 2013) as implemented in USEARCH 10.0.240. Higher taxonomic levels were assigned using the RDP classifier version 2.13 at a cutoff bootstrap confidence of 80% (Wang et al. 2007). Reads that were identified as typical contaminants using blanks and correlation analysis (Szafrański et al. 2015) were removed.

The functional potential of sample groups was predicted from the full-16S-based composition using PICRUSt2 (v2.5.2) (Douglas et al. 2020). PICRUSt2 was applied with default settings and nearest-sequenced taxon index (NSTI) set to 3. This allowed us to include 3 three SR1 taxa that are underrepresented in the database and have no closely related genome in the database.

For RNA sequencing data analysis, BCL files were converted into FASTQ files (single-end reads) using the bcl2fastq software version v2.20.0.422 (Illumina). Quality control of sequencing reads was performed with *fastp* v0.20.1, an ultra-fast all-in-one FASTQ preprocessor (Chen et al. 2018). Reads originating from human sources were removed using a mapping approach that utilized *bwa-samse* (version 0.7.17, build 0.7.17-r118866) (Li and Durbin 2009) against the GRCh38.p13 human reference genome (GCA_000001405.28, NCBI RefSeq, PRJNA31257, maintained by the Genome Reference Consortium).

To construct a metagenome reference specific to the human oral cavity, sequences from two publicly available datasets were merged. The first source was the expanded Human Oral Microbiome Database (eHOMD, Rev: 2021-10-01), which contains 2,087 bacterial genomes representing microorganisms from the human mouth and aerodigestive tract (Escapa et al. 2018). The second source (Pasolli et al. 2019), which provided 9,428 metagenomes and 154,723 metagenome-assembled genomes (MAGs) grouped into species-level genome bins (SGBs). From this study, 7,897 MAGs identified as originating from oral-cavity-derived samples were specifically selected. Sequences from these two datasets were combined into a unified oral metagenome reference set. The sequences were grouped into species-level taxonomic units (TUs) based on 95% average nucleotide identity (ANI) using the Pyani standalone program version pyani 0.2.11 and annotated using the *Prokka* pipeline v1.14.6 (Seemann 2014). Additional EC numbers were assigned to these annotations using the eggNOG orthology database (Huerta-Cepas et al. 2019) using eggNog-mapper: emapper-2.1.6 instance, extending the functional information.

The combined reference database incorporated original taxonomy annotations from eHOMD, enhanced by *Prokka* and eggNOG annotations, providing a robust resource for both taxonomy and functional insights. To refine the database further, taxa not represented in the patient cohort were excluded. This was achieved by mapping oral metagenome sample reads to the combined database using *BWA-samse* (Danecek et al. 2021; Escapa et al. 2018) and retaining only those TUs sufficiently covered by sample reads. Additionally, rRNA-coding genes were excluded from the final mapping based on annotation.

For transcriptomic analysis, sample reads were mapped to protein-coding gene sequences within the selected TUs (SGBs) of the customized oral metagenome reference set using *BWA-samse*. To prevent bias in transcript abundance calculations, reads mapped to multiple features were randomly distributed among them. Read counts were determined using the *htseq-count* program from the HTSeq package v0.13.5 (Anders et al. 2015). To simplify the data and focus on functional features, counts were aggregated based on gene-EC relationships. For genes associated with multiple EC numbers, counts were grouped at the highest shared EC hierarchy level. When discrepancies occurred at the second-highest level, the reads were assigned to an "Unknown" category.

***Statistical analysis***

Good’s coverage index (Good, 1953) was calculated for each sample across both species/OTU (99.16 ± 0.005; mean ± 95% CI) and EC datasets (99.98 ± 0.001; mean ± 95% CI), considering singletons and low abundance features (n_r_ < 6), where n_r_ refers to the number of distinct species/OTUs or ECs observed fewer than six times in the sample. We selected this stricter threshold (< 6) to account for the high throughput and sequencing depth of the methods used. Microbial α-diversity was analyzed using the Chao1 and Shannon indices. Bray-Curtis distances calculated from square root-transformed relative abundances of microbial and functional data were visualized with nMDS ordinations. Multivariate analysis of microbial taxa, and predicted functional pathways was performed using PRIMER 7 (Clarke and Gorley 2015) with PERMANOVA+ (Anderson and Gorley 2008). PERMANOVA with ‘patient’ as a random factor with 9999 permutations was used to test the differences between two groups of peri-implantitis severities generated using increasing PD cut-offs. PERMANOVA was modelled with ‘PD’ as a fixed factor, followed by ‘patient’ as a random factor, with Type III (partial) Sums of Squares and permutations under a reduced model. PERMDISP was used to assess within-group dispersion. BEST (BIO-ENV) routine was used for matching multivariate microbial patterns with clinical parameters and their combinations. For the PERMDISP and BIO-ENV analyses, random factors could not be incorporated into the model. To account for intra-patient similarity, we randomly selected one implant per patient for these analyses. DESeq2 v1.44.0 (Love et al. 2014) analysis was used to identify significant (Benjamini Hochberg correction - adjusted-*p* < 0.05, Log_2_fold change > 2.0) associations of individual microbial taxa with PD as categorical variable (two groups). Microbiome Multivariable Associations with Linear Models (MaAsLin2) (v1.20.0) package (Mallick et al. 2021) was used to identify associations of microbial taxa, predicted functions and RNAseq ECs with PD as continuous variable and categorical variable with ‘patient’ as random effect. The default MaAsLin2 adjusted-*p* < 0.25 cut-off was considered significant. To control for smokers and age, samples were excluded (n = 8) and the statistical model was expanded, respectively. Biomarker genera, *i.e.*, *Gemella*, *Capnocytophaga* and *Pseudoramibacter* remained significant in non-smoking populations and after adjusting for age as a covariate (MaAsLin2 FDR *p* < 0.25 for both). Due to the smaller size of the metatranscriptomic dataset, we limited our model to PD as the sole fixed covariate and patient as a random covariate, as including additional variables risked overfitting and reduced model stability.

***Calculation of MDI and eMDI for peri-implantitis severity***

A microbial dysbiosis index (MDI) for peri-implantitis severity was calculated using 3 MaAsLin2-based significant genera as in Gevers et al. 2014. An extended MDI (eMDI) for peri-implantitis severity was proposed, that combined the abundances of genus-level taxa and RNAseq ECs and was determined by sequential addition of features.

MDI was calculated based on following:

$MDI=\mathrm{Log}_{10}\left[ \frac{Genera positively associated with PD}{Genera negatively associated with PD} \right]$, where values were relative abundances.

In our study, we included abundances of 3 MaAsLin2-based genera significantly associated with PD, as follows: $MDI=\mathrm{Log}_{10}\left[ \frac{Pseudoramibacter}{Capnocytophaga+ Gemella} \right]$

Finally, Extended-MDI (eMDI) for peri-implantitis severity was calculated by integrating RNAseq enzyme features in MDI:

$eMDI=\mathrm{Log}_{10}\left[ \frac{Genera and ECs positively associated with PD}{Genera and ECs negatively associated with PD} \right]$, where values were scaled so that their maximum value across samples is always 100.

Feature reduction of negatively associated ECs was achieved through a stepwise approach. The eMDI was tested multiple times by sequential addition of negatively associated ECs, starting from 1 to all MaAsLin2 significant ECs. The eMDI in our study was calculated as follows:

$$\mathrm{eMDI}=\mathrm{Log}_{10}\left[ \frac{Pseudoramibacter + 3.4.23.36 + 3.4.21.53 + 3.4.21.102}{Capnocytophaga + Gemella + 2.7.1.204 + 4.1.2.40 + 5.3.1.26 + 2.7.1.144 + 3.2.1.20 + 1.12.99.6} \right]$$

The performance of MDI and eMDI was assessed using area under the receiver’s operating characteristic curve (AUC-ROC). Additionally, to assess the multicollinearity or redundancy between the features included in eMDI, hierarchical clustering and correlation analysis was performed.

**Appendix Discussion**

*Pseudoramibacter* showed significantly positive correlation with PD in our cohort. This is in agreement with the findings from a previous German-Swedish peri-implantitis severity cohort, where the corresponding provisional family [*Eubacteriaceae XV*], to which *Pseudoramibacter* belongs, was also identified as the most prominent marker of severe disease (Kröger et al. 2018). We re-examined Cluster XV, previously reported to include ten species ([WILLEMS and COLLINS, 1996](#_ENREF_1)), of which only *Pseudoramibacter alactolyticus* is a confirmed oral species (eHOMD, accessed June 2025). The other nine, including *Eubacterium limosum,* which is defined as of unknown primary site, are mainly associated with the gut or environmental sources. The striking convergence of evidence across two independent populations firmly establishes *Pseudoramibacter* as a robust and reproducible biomarker of peri-implantitis severity, highlighting its potential utility in clinical diagnostics. In a recent metagenomic study, *P. alactolyticus* was found to be 10 times more enriched in peri-implantitis than in healthy sites (Ghensi et al. 2020) and has been linked to late implant loss (Korsch et al. 2021). Interestingly, in the largest U.S. based study comprising over 18,000 isolates, the incidence of *Pseudoramibacter* in subgingival plaque nearly doubled in severe periodontitis compared to moderate forms, while remaining low or absent in health, further reinforcing its association with advanced oral inflammatory pathology (Moore et al. 1985). Another periodontal study demonstrated an increased frequency of *P*. *alactolyticus* in refractory cases as compared to good responders to therapy (Colombo et al. 2012). Whether *Pseudoramibacter* actively drives disease progression or preferentially colonizes compromised tissues remains an open question. We hypothesize that fastidious *Pseudoramibacter* serves as an indicator of prolonged dysbiosis, rather than being directly involved in the destructive process itself. Given its apparent ability to integrate clinical disease information over extended periods, *Pseudoramibacter* may represent a valuable biomarker for patient stratification, with higher abundance potentially indicating the need for more intensive therapeutic intervention.

PICRUSt2-based analyses revealed functional shifts associated with disease severity. The differences in microbial activity observed across pocket depths likely reflect a progressive shift in the submucosal environment, from a more aerated, higher redox state with greater salivary nutrient input, to a more anaerobic, reduced environment increasingly influenced by mucosal crevicular fluid and nutrients derived from host tissue breakdown (Takahashi 2015). Specifically, in shallower pockets, salivary glycoproteins and dietary nitrate (concentrated in salivary glands) support central carbon metabolism and nitrate respiration, respectively. The observed reduction in heme biosynthesis in deeper pockets may reflect the metabolic strategy of key pathogens such as *Porphyromonas gingivalis*, which scavenge heme from the host or from cohabiting microbes rather than synthesizing it *de novo* (Olczak et al. 2005). In contrast, the increased abundance of cobalamin (vitamin B12) and tetrahydrofolate (THF) biosynthesis pathways with pocket depth suggests a heightened demand and relative scarcity of these essential cofactors in the peri-implantitis microenvironment of deeper pockets. As central players in one-carbon metabolism, DNA synthesis, and methionine biosynthesis (Rodionov et al. 2003, Maden 2000), these cofactors likely become limiting during biofilm expansion, and de novo biosynthesis may be critical to support biofilm proliferation where environmental scavenging is insufficient.

**Appendix References**

Anders S, Pyl PT, Huber W. 2015. HTSeq--a Python framework to work with high-throughput sequencing data. *Bioinformatics*. 31(2):166-9.

Anderson M, Gorley RN, Clarke K. 2008. PERMANOVA+ for primer: Guide to software and statistical methods.

Armitage GC, Xenoudi P. 2016. Post-treatment supportive care for the natural dentition and dental implants. *Perio 2000*. 71(1):164-184.

Berglundh T, Armitage G, Araujo MG, Avila-Ortiz G, Blanco J, Camargo PM, Chen S, Cochran D, Derks J, Figuero E, et al. 2018. Peri-implant diseases and conditions: Consensus report of workgroup 4 of the 2017 World Workshop on the Classification of Periodontal and Peri-Implant Diseases and Conditions. J Periodontol. 89(Suppl 1):S313-S318.

Colombo AP, Haffajee AD, Dewhirst FE, Paster BJ, Smith CM, Cugini MA, Socransky SS. 1998. Clinical and microbiological features of refractory periodontitis subjects. *J Clin Periodontol*. 25(2):169-80.

Chen S, Zhou Y, Chen Y, Gu J. 2018. fastp: an ultra-fast all-in-one FASTQ preprocessor. *Bioinformatics*. 34(17):i884-i890.

Chen T, Yu WH, Izard J, Baranova OV, Lakshmanan A, Dewhirst FE. 2010. The Human Oral Microbiome Database: a web accessible resource for investigating oral microbe taxonomic and genomic information. *Database (Oxford)*. 2010:baq013.

Clarke K, Gorley RN. 2015. PRIMER version 7: User manual/tutorial. PRIMER-E 192.

Danecek P, Bonfield JK, Liddle J, Marshall J, Ohan V, Pollard MO, Whitwham A, Keane T, McCarthy SA, Davies RM, et al. 2021. Twelve years of SAMtools and BCFtools. *Gigascience*. 10(2):giab008.

Dieckow S, Szafrański SP, Grischke J, Qu T, Doll-Nikutta K, Steglich M, Yang I, Häussler S, Stiesch M. 2024. Structure and composition of early biofilms formed on dental implants are complex, diverse, subject-specific and dynamic. *npj Biofilms Microbiomes*.10(1):155.

Douglas GM, Maffei VJ, Zaneveld JR, Yurgel SN, Brown JR, Taylor CM, Huttenhower C, Langille MGI. 2020. PICRUSt2 for prediction of metagenome functions. *Nat Biotechnol.* 38(6):685-688.

Edgar RC. 2013. UPARSE: highly accurate OTU sequences from microbial amplicon reads. *Nat Methods.* 10(10):996-8.

Escapa IF, Chen T, Huang Y, Gajare P, Dewhirst FE, Lemon KP. 2018. New insights into human nostril microbiome from the expanded human oral microbiome database (eHOMD): a resource for the microbiome of the human aerodigestive tract. *mSystems*. 3(6):e00187-18.

Ganesan SM, Dabdoub SM, Nagaraja HN, Mariotti AJ, Ludden CW, Kumar PS. 2022. Biome-microbiome interactions in peri-implantitis: A pilot investigation. *J Periodontol*. 93(6):814-823.

Gevers D, Kugathasan S, Denson LA, Vázquez-Baeza Y, Van Treuren W, Ren B, Schwager E, Knights D, Song SJ, Yassour M, et al. 2014. The treatment-naive microbiome in new-onset Crohn's disease. *Cell Host Microbe*. 15(3):382-392.

Ghensi P, Manghi P, Zolfo M, Armanini F, Pasolli E, Bolzan M, Bertelle A, Dell'Acqua F, Dellasega E, Waldner R, et al. 2020. Strong oral plaque microbiome signatures for dental implant diseases identified by strain-resolution metagenomics. *NPJ Biofilms Microbiomes*. 6(1):47.

Grischke J, Szafrański SP, Muthukumarasamy U, Haeussler S, Stiesch M. 2021.Removable denture is a risk indicator for peri-implantitis and facilitates expansion of specific periodontopathogens: a cross-sectional study. *BMC Oral Health*. 21(1):173.

Huerta-Cepas J, Szklarczyk D, Heller D, Hernández-Plaza A, Forslund SK, Cook H, Mende DR, Letunic I, Rattei T, Jensen LJ, et al. 2019. eggNOG 5.0: a hierarchical, functionally and phylogenetically annotated orthology resource based on 5090 organisms and 2502 viruses. *Nucleic Acids Res*. 47(D1):D309-D314.

Korsch M, Marten SM, Stoll D, Prechtl C, Dötsch A. 2021. Microbiological findings in early and late implant loss: an observational clinical case-controlled study. *BMC Oral Health*. 21(1):112.

Kröger A, Hülsmann C, Fickl S, Spinell T, Hüttig F, Kaufmann F, Heimbach A, Hoffmann P, Enkling N, Renvert S, et al. 2018. The severity of human peri-implantitis lesions correlates with the level of submucosal microbial dysbiosis. *J Clin Periodontol*. 45(12):1498-1509.

Lange DE, Plagmann HC, Eenboom A, Promesberger A. 1977. Klinische Bewertungsverahren zur Objektivierung der Mund hygiene [Clinical methods for the objective evaluation of oral hygiene]. *Dtsch Zahnarztl Z*. 32(1):44-7.

Li H, Durbin R. 2009. Fast and accurate short read alignment with Burrows-Wheeler transform. *Bioinformatics*. 25(14):1754-60.

Love MI, Huber W, Anders S. 2014. Moderated estimation of fold change and dispersion for RNA-seq data with DESeq2. *Genome Biol*. 15:550.

Maden BE. 2000. Tetrahydrofolate and tetrahydromethanopterin compared: functionally distinct carriers in C1 metabolism. *Biochem J.* 350(3):609-29.

Mallick H, Rahnavard A, McIver LJ, Ma S, Zhang Y, Nguyen LH, Tickle TL, Weingart G, Ren B, Schwager EH, et al. 2021. Multivariable association discovery in population-scale meta-omics studies*. PLoS Comput Biol*. 17(11):e1009442.

Moore WE, Moore LH, Ranney RR, Smibert RM, Burmeister JA, Schenkein HA. 1991. The microflora of periodontal sites showing active destructive progression. *J Clin Periodontol*. 18(10):729-39.

Moore WE, Holdeman LV, Cato EP, Smibert RM, Burmeister JA, Palcanis KG, Ranney RR. 1985. Comparative bacteriology of juvenile periodontitis. *Infect Immun*. 48(2):507-19.

Olczak T, Simpson W, Liu X, Genco CA. 2005. Iron and heme utilization in Porphyromonas gingivalis. *FEMS Microbiol Rev.* 29(1):119-44.

Pasolli E, Asnicar F, Manara S, Zolfo M, Karcher N, Armanini F, Beghini F, Manghi P, Tett A, Ghensi P, et al. 2019. Extensive unexplored human microbiome diversity revealed by over 150,000 genomes from metagenomes spanning age, geography, and lifestyle. *Cell*. 176(3):649-662.e20.

Quast C, Pruesse E, Yilmaz P, Gerken J, Schweer T, Yarza P, Peplies J, Glöckner FO. 2013. The SILVA ribosomal RNA gene database project: improved data processing and web-based tools. *Nucleic Acids Res*. 41(Database issue):D590-6.

Rodionov DA, Vitreschak AG, Mironov AA, Gelfand MS. 2003. Comparative genomics of the vitamin B12 metabolism and regulation in prokaryotes. *J Biol Chem.* 278(42):41148-59.

Seemann T. 2014. Prokka: rapid prokaryotic genome annotation. *Bioinformatics*. 30(14):2068-9.

Shiba T, Watanabe T, Kachi H, Koyanagi T, Maruyama N, Murase K, Takeuchi Y, Maruyama F, Izumi Y, Nakagawa I. 2016. Distinct interacting core taxa in co-occurrence networks enable discrimination of polymicrobial oral diseases with similar symptoms. *Sci Rep.* 6:30997.

Solbiati J, Frias-Lopez J. 2018. Metatranscriptome of the Oral Microbiome in Health and Disease. *J Dent Res*. 97(5):492-500.

Szafranski SP, Wos-Oxley ML, Vilchez-Vargas R, Jáuregui R, Plumeier I, Klawonn F, Tomasch J, Meisinger C, Kühnisch J, Sztajer H, et al. 2015. High-resolution taxonomic profiling of the subgingival microbiome for biomarker discovery and periodontitis diagnosis. *Appl Environ Microbiol*. 81(3):1047-58.

Takahashi N. 2015. Oral microbiome metabolism: from “who are they?” to “what are they doing?”. *J Dent Res*. 94(12):1628-1637.

Wang Q, Garrity GM, Tiedje JM, Cole JR. 2007. Naive Bayesian classifier for rapid assignment of rRNA sequences into the new bacterial taxonomy. *Appl Environ Microbiol*. 73(16):5261-7.

Whittaker RH. 1972 Evolution and measurement of species diversity. *Taxon*. 21(2-3):213-51.

WILLEMS A, COLLINS MD (1996). Phylogenetic Relationships of the Genera Acetobacterium and Eubacterium Sensu Stricto and Reclassification of Eubacterium alactolyticum as Pseudoramibacter alactolyticus gen. nov., comb. nov. *International Journal of Systematic and Evolutionary Microbiology* 46(4):1083-1087.

Yarza P, Richter M, Peplies J, Euzeby J, Amann R, Schleifer KH, Ludwig W, Glöckner FO, Rosselló-Móra R. 2008. The All-Species Living Tree project: a 16S rRNA-based phylogenetic tree of all sequenced type strains. *Syst Appl Microbiol*. 31(4):241-50.

| Inclusion Criteria | Exclusion Criteria |
| --- | --- |
| - Patients older than 18 years of age - Signed informed consent form - Systemically healthy patients with presence of at least one implant with the diagnosis of peri-implantitis - Peri-implantitis diagnosed implant(s) restored with prostheses for at least one year | - Presence of periodontal disease - Presence of chronic systemic disease (*e.g.*, cardiovascular disease, chronic kidney disease, autoimmune disease, cancer, chronic respiratory disease) - Use of medications known to affect salivary flow (*e.g*., immunosuppressant medications, bisphosphonates, or corticosteroids) - Use of antimicrobials, antibiotics and anti-inflammatory medication within past 6 months - Pregnancy or lactation - Heavy smokers (>20 cigarettes/day) - Participants who have received prior treatment for peri-implant disease with the same implant, except for supragingival prophylaxis - Participants with a history of periodontitis less than 2 years prior to implant placement |

**Appendix Table 1** Selection criteria for study participants

| **Demographic Characteristics** | | |
| --- | --- | --- |
| **Number of patients** | | 34 |
| **Number of implants** (n) | | 49 |
| **Population-specific parameters** | | |
| **Patient age in years** (Mean ± SD) |  | 73 ± 8.9 |
| **Patient sex** (%) | Males | 50 |
|  | Females | 50 |
| **Smoking** (%) | Yes | 5.9 |
|  | No | 88.2 |
|  | n/a | 5.9 |
| **API** (%) | ≤ 35% | 38.2 |
|  | > 35% | 61.8 |
| **Implant-specific parameters** | | |
| **Gingival index** (Mean ± SD) |  | 2.2 ± 0.8 |
| **Plaque index** (Mean ± SD) |  | 1.9 ± 1.1 |
| **Implant position** (%) | Incisor | 20.4 |
|  | Cuspid | 14.3 |
|  | Bicuspid | 28.6 |
|  | Molar | 36.7 |
| **Years of implant in function** (Mean ± SD) |  | 9.8 ± 6.1 |
| **Bleeding on probing** (%) | Yes | 100 |
|  | No | 0 |
| **Pocket depth** (Mean ± SD) |  | 7.1 ± 2.3 |
| **Suppuration** (%) | Yes | 40.82 |
|  | No | 59.18 |
| **Periotron** (Mean ± SD) |  | 126.9 ± 47.4 |
| **Pain** (%) | Yes | 24.5 |
|  | No | 75.5 |

**Appendix Table 2** Population-specific and implant-specific characteristics of the study population. API – Approximal Plaque Index (Lange 1977)

| **EC no.** | **Enzyme name** | **Regression co-efficient** | **Std Error** | ***p* value** | **Adj *p*** |
| --- | --- | --- | --- | --- | --- |
| 4.2.2.7 | heparin lyase | -1.907 | 0.453 | 0.0001 | 0.067 |
| 4.2.2.8 | heparin-sulfate lyase | -1.907 | 0.453 | 0.0001 | 0.067 |
| 3.5.1.11 | penicillin amidase | -1.263 | 0.299 | 0.0001 | 0.067 |
| 2.7.7.68 | 2-phospho-L-lactate guanylyltransferase | -1.253 | 0.303 | 0.0002 | 0.079 |
| 3.1.2.20^§^ | acyl-CoA hydrolase | -1.298 | 0.330 | 0.0003 | 0.095 |
| 3.5.1.5 | urease | -1.244 | 0.342 | 0.0007 | 0.143 |
| 1.2.1.21^§^ | glycolaldehyde dehydrogenase | -1.089 | 0.308 | 0.0010 | 0.143 |
| 1.2.1.22^§^ | lactaldehyde dehydrogenase | -1.089 | 0.308 | 0.0010 | 0.143 |
| 3.1.2.28^§^ | 1,4-dihydroxy-2-naphthoyl-CoA hydrolase | -1.080 | 0.284 | 0.0006 | 0.143 |
| 1.7.2.1 | nitrite reductase (NO-forming) | -0.766 | 0.220 | 0.0011 | 0.143 |
| 1.1.1.31 | 3-hydroxyisobutyrate dehydrogenase | -0.333 | 0.089 | 0.0010 | 0.143 |
| 4.1.2.25 | dihydroneopterin aldolase | 0.065 | 0.016 | 0.0011 | 0.143 |
| 3.2.1.170 | mannosylglycerate hydrolase | -1.510 | 0.444 | 0.0015 | 0.175 |
| 1.1.1.284 | S-(hydroxymethyl)glutathione dehydrogenase | -1.312 | 0.417 | 0.0029 | 0.244 |
| 1.1.1.339 | dTDP-6-deoxy-L-talose 4-dehydrogenase (NAD+) | -0.973 | 0.304 | 0.0027 | 0.244 |
| 1.5.1.38 | FMN reductase (NADPH) | -0.708 | 0.221 | 0.0029 | 0.244 |
| 1.2.1.18 | malonate-semialdehyde dehydrogenase (acetylating) | -0.705 | 0.221 | 0.0029 | 0.244 |
| 1.2.1.27 | methylmalonate-semialdehyde dehydrogenase (CoA-acylating) | -0.705 | 0.221 | 0.0029 | 0.244 |
| 3.5.1.23^£^ | Ceramidase | -1.594 | 0.346 | 0.000 | 0.109 |
| 2.4.1.109^£^ | dolichyl-phosphate-mannose--protein mannosyltransferase | -1.530 | 0.397 | 0.001 | 0.234 |

**Appendix Table 3** PICRUSt2 based functional ECs significantly correlated with PD (adjusted-*p* < 0.25) across samples (n = 49). A positive regression coefficient indicates a positive correlation with PD, while a negative coefficient indicates an inverse correlation. § denotes ECs that were also significantly associated with PD in the RNA-seq matched dataset (n = 27). £ denotes additional ECs that were not significant in the original dataset (n = 49) but were significant in the RNA-seq matched dataset (n = 27).


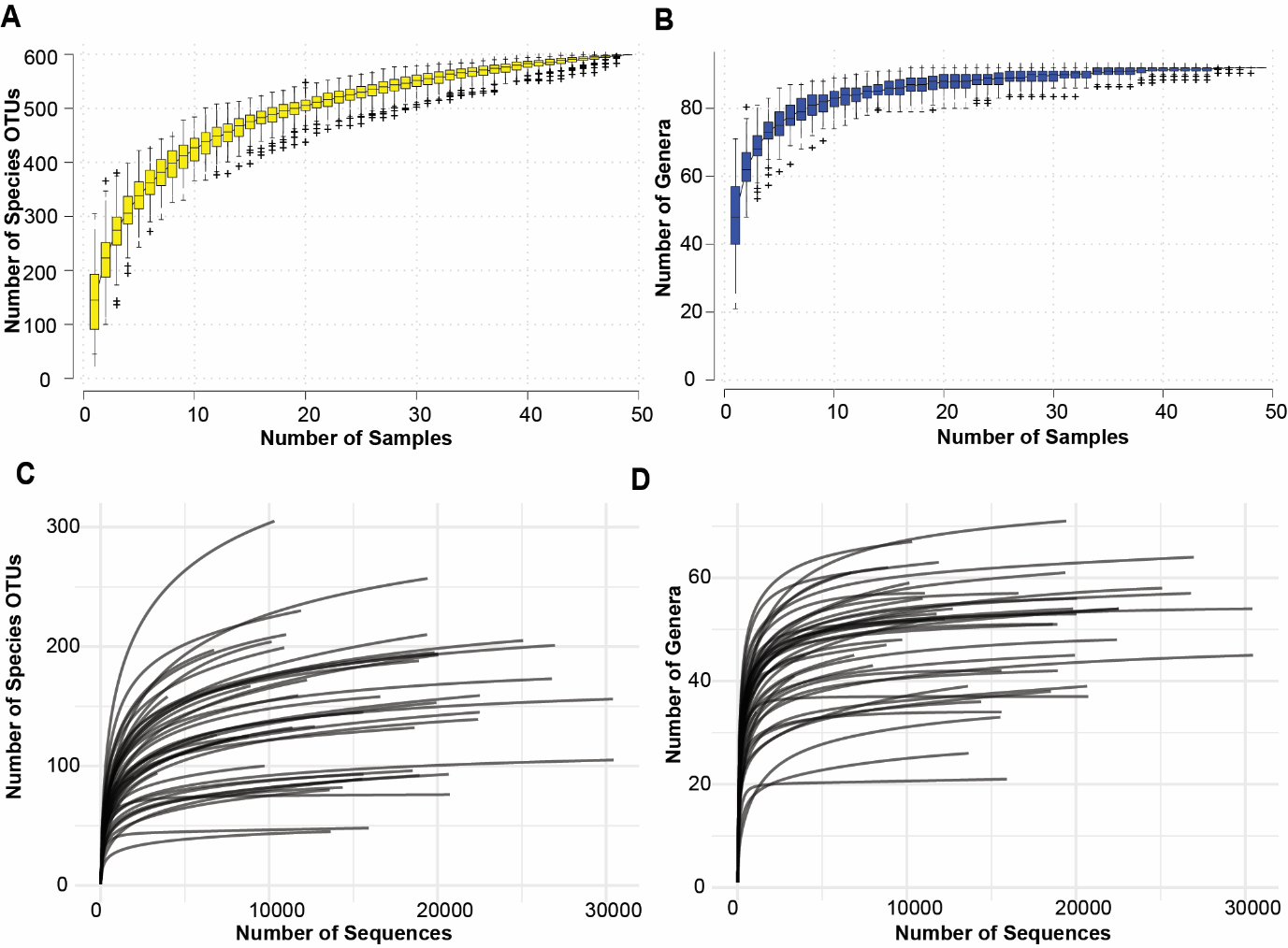


**Appendix Figure 1**

Microbial diversity across peri-implantitis samples. **(A)** Species accumulation curve and **(B)** genus accumulation curve, with the x-axis representing the number of samples and the y-axis showing the cumulative number of observed species or genera, respectively. **(C)** Rarefaction curves per sample at the species level and **(D)** at the genus level, illustrating OTU-based diversity for each sample.


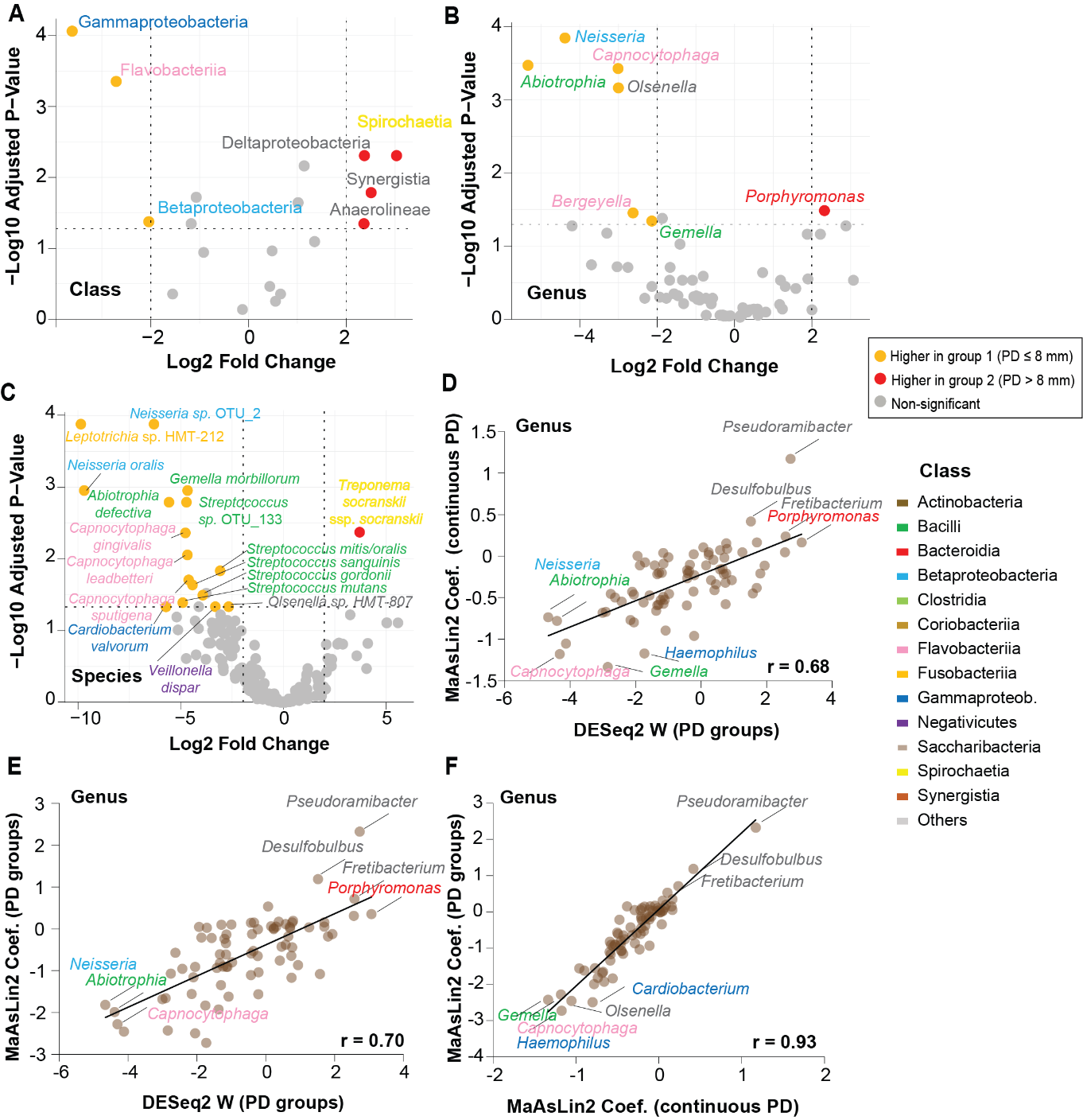


**Appendix Figure 2**

Volcano plots of DESeq2-based differential abundances of microbial **(A)** classes, **(B)** genera and **(C)** species between group 1 and group 2 peri-implantitis samples not adjusted for patients as random effect (Log2Fold change cut-off > 2; adjusted-*p* value < 0.05; BaseMean cut-off > 5). Dot plots showing the Pearson’s correlation between the MaAsLin regression coefficient and DESeq2 Wald statistic of genus level data **(D)** DESeq2 (PD groups) and MaAsLin2 (continuous PD), **(E)** DESeq2 (PD groups) and MaAsLin2 (PD groups) and **(F)** MaAsLin2 (continuous PD) and MaAsLin2 (PD groups).


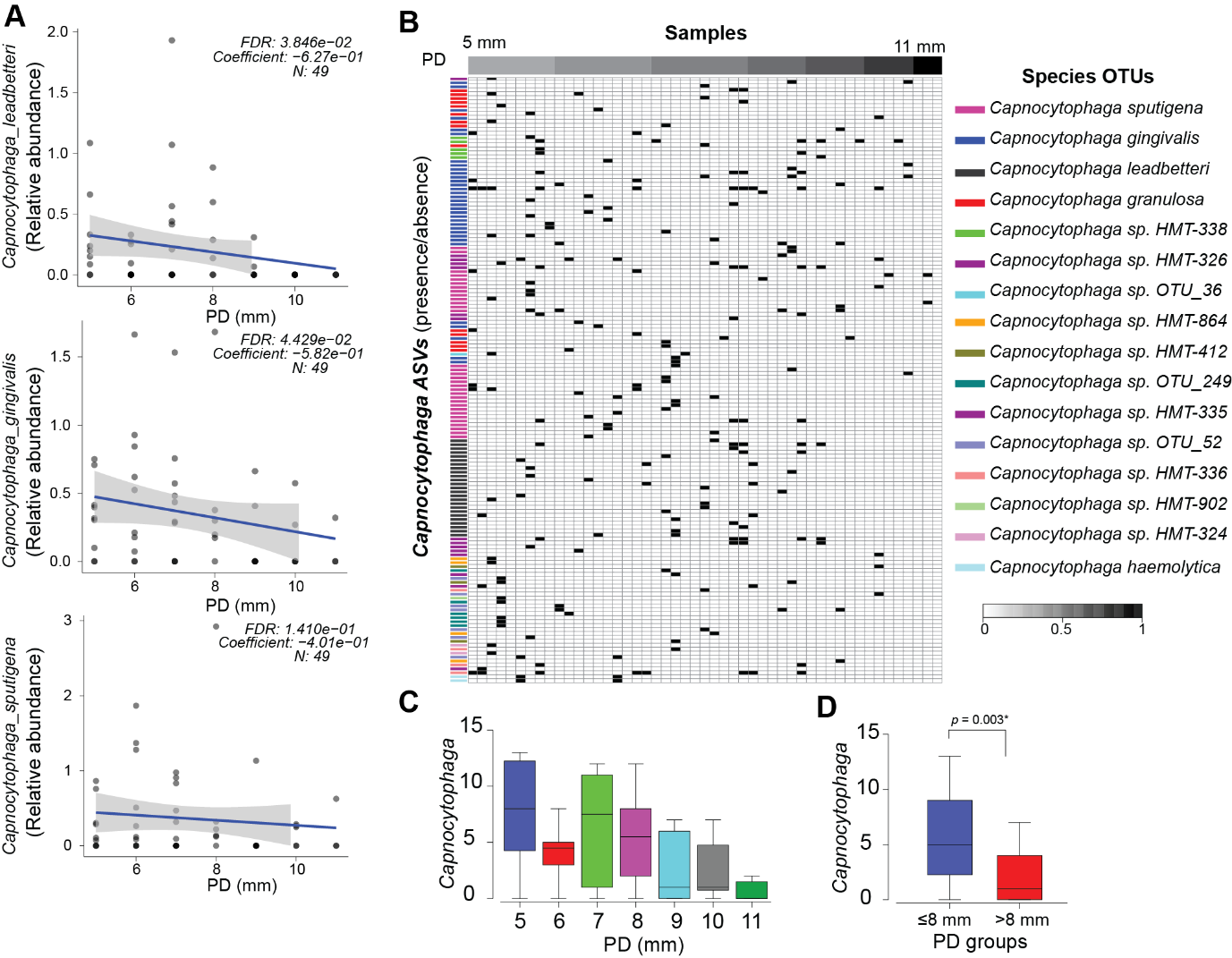


**Appendix Figure 3**

Subgenus level analysis of biomarker *Capnocytophaga* genus **(A)** Scatterplots of main *Capnocytophaga* species with PD. All regression correlations were calculated with ‘patient’ as a random effect variable. **(B)** Heatmap for Amplicons sequencing variance (ASVs) for *Capnocytophaga* species. Samples sorted by increasing PD. **(C)** Number of *Capnocytophaga* ASVs per sample averaged for PD. (**D**) Number of *Capnocytophaga* ASVs per sample averaged for PD groups.


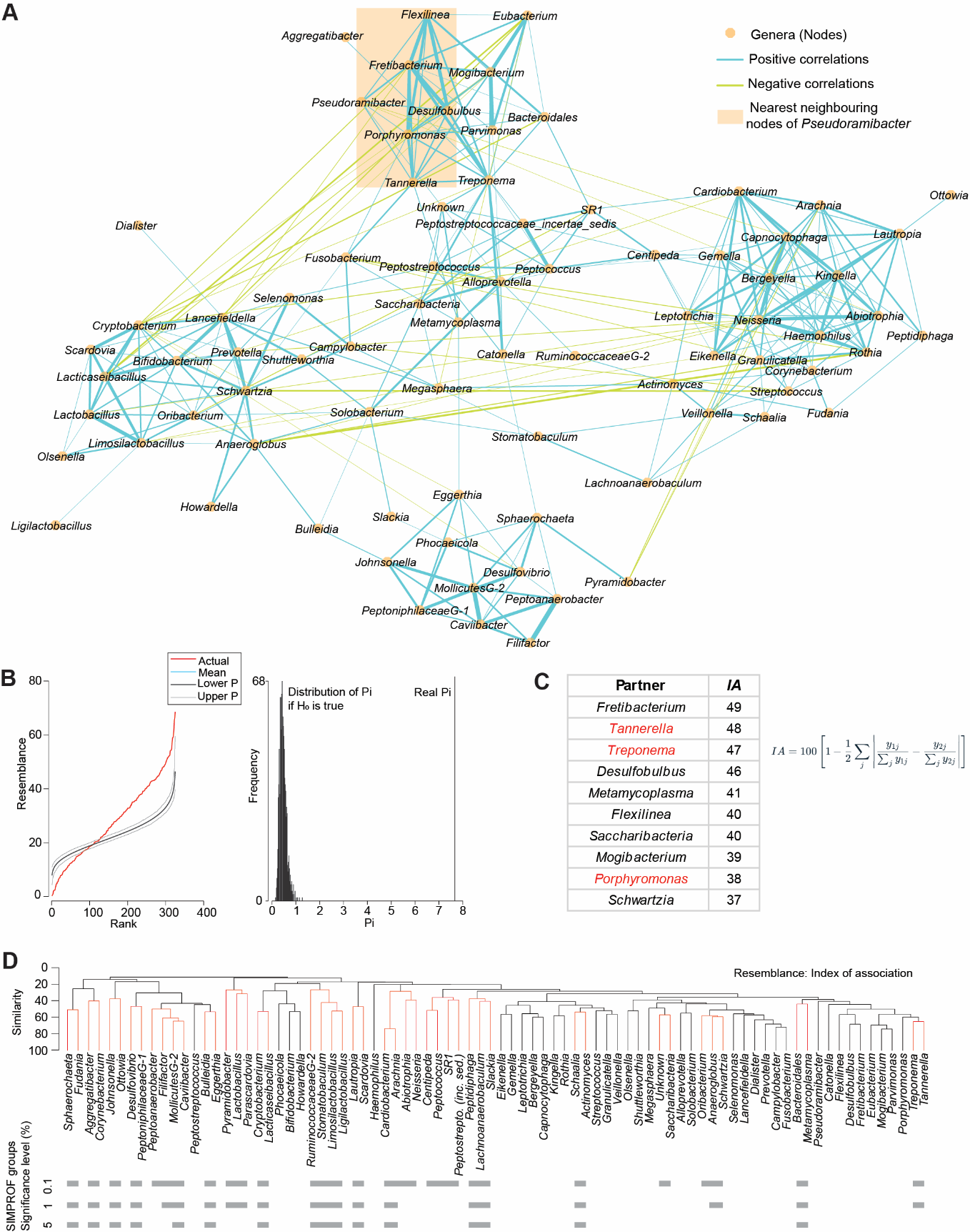


**Appendix Figure 4**

Relationship of *Pseudoramibacter* with other genera. **(A)** Network analysis of bacterial genera with a relative abundance of at least 0.1% across all samples. Edges represent significant pairwise associations based on Spearman rank correlations with |r| greater than 0.4. Blue lines indicate positive correlations while green lines indicate negative correlations between genera. Weights vary between -0.66 to -0.4 and 0.4 to 0.86. The shaded area represents the nearest neighboring genera positively correlated with *Pseudoramibacter*. **(B)** Multivariate measure of genera co-occurrence based on similarity profiles calculated with R-mode analysis using pairwise Whittaker’s associations for genera **(C)** *IA* scores for combinations of *Pseudoramibacter* and its top 10 associated genera **(D)** Dendogram (agglomerative, group average linked) calculated from an index of association matrix among genera. Red lines and grey boxes denote the coherent groups of genera with different significance levels set for SIMPROF tests (5%, 1% and 0.1%) to discover stable relationships. Type 2 SIMPROF tests were performed to validate the hypothesis that there are no associations of any sort among taxa. Values were permuted 100,000 times for each taxon across all samples. *IA* – Index of association between Bray-Curtis similarities based on standardized variables, with 0 implying full ‘negative’ and 100 full ‘positive’ association of the two taxa (Whittaker 1972).


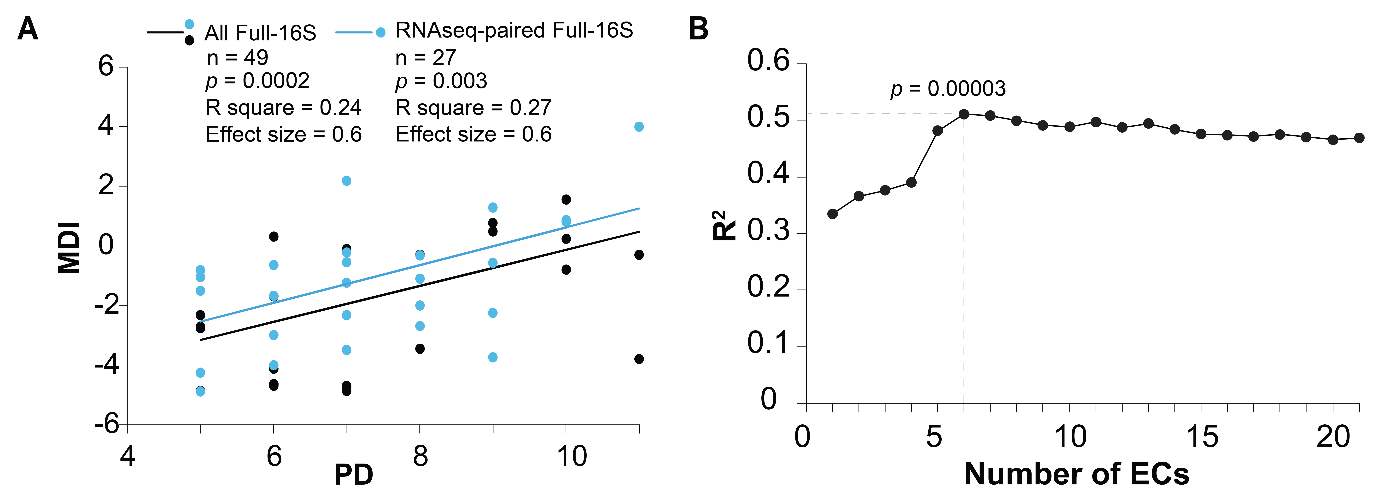


**Appendix Figure 5**

**(A)** Scatterplot of association of genus-level MDI with PD for both original (n = 49) and RNAseq matched (n = 27) datasets. Linear regression lines fitted separately for each group. **(B)** Relationship between the number of negatively associated ECs included in the calculation of eMDI for peri-implantitis severity and the corresponding R^2^ values.


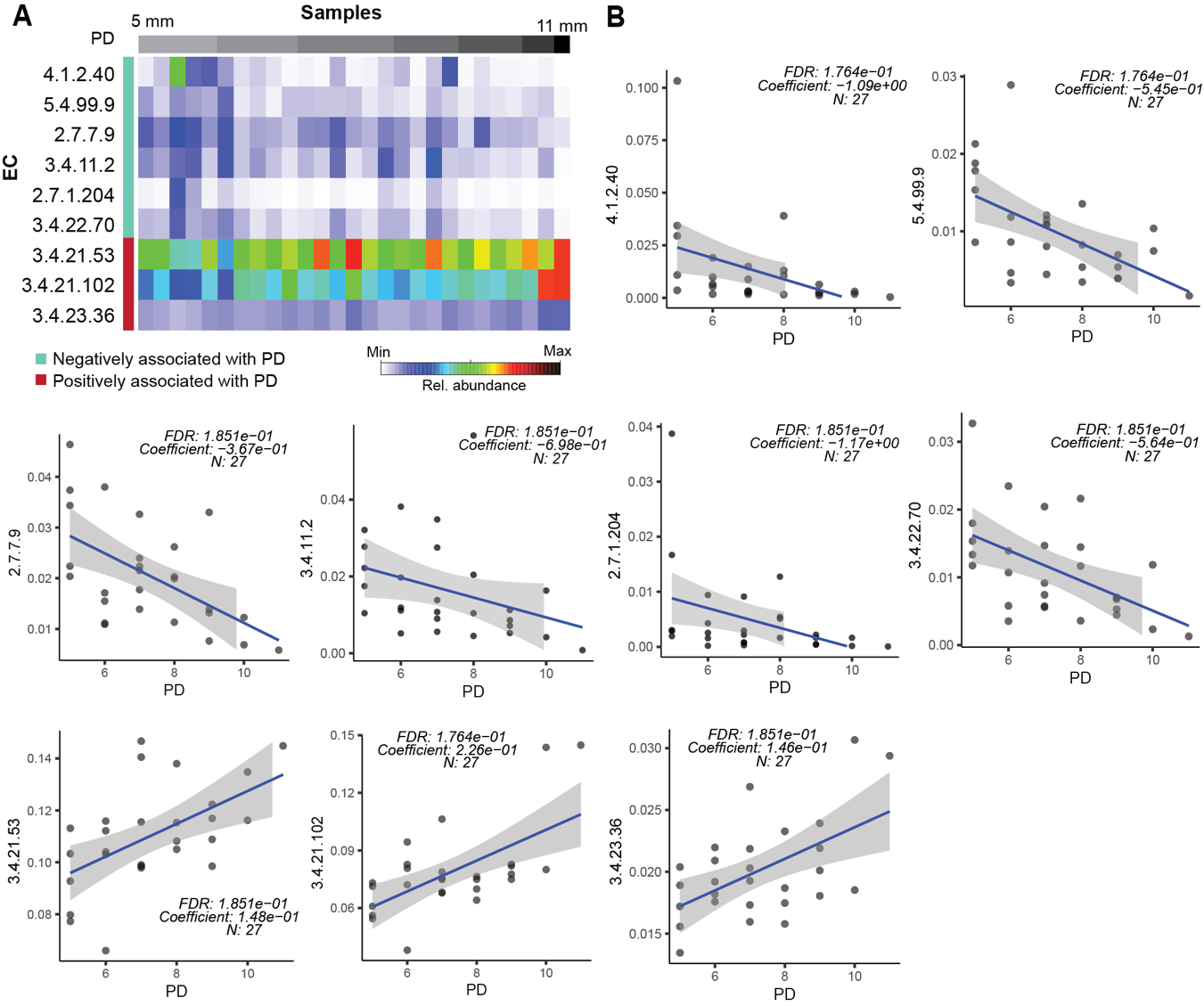


**Appendix Figure 6**

**(A)** Heatmap showing the relative abundances of RNAseq ECs (MaAsLin2 default adjusted-*p* < 0.25) included in eMDI across samples sorted by increasing PD. **(B)** Scatterplots of ECs with the highest significant correlations to changes in PD and those included in eMDI. All regression correlations were calculated with ‘patient’ as a random effect variable.


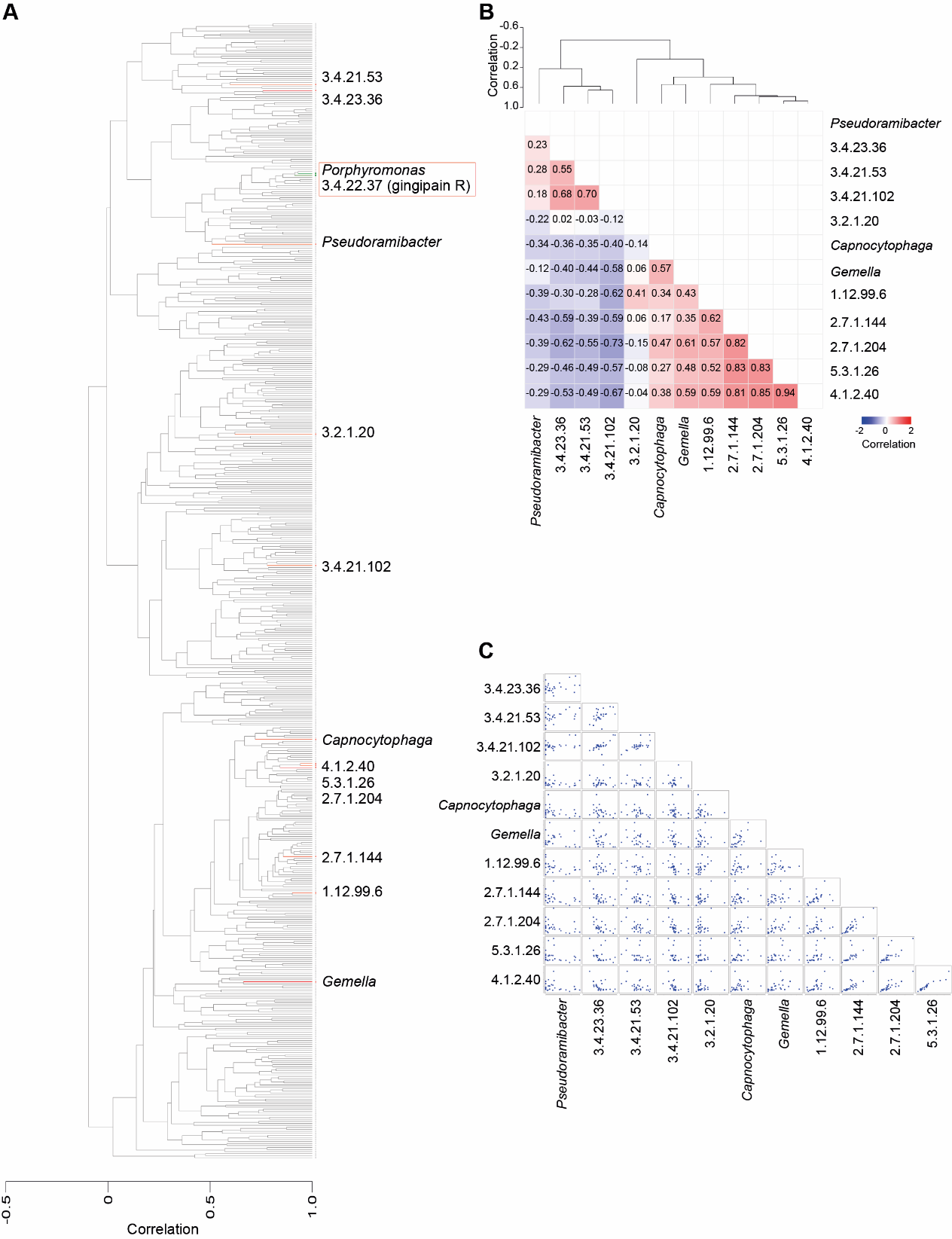


**Appendix Figure 7**

**(A)** Hierarchical clustering of selected 16S genera and RNAseq ECs (≥ 0.1% abundance across samples) using Spearman correlation and average linkage. *Porphyromonas* and gingipain R association highlighted as expected redundancy. **(B)** Spearman correlation heatmap of taxa and EC features included in the eMDI. **(C)** Draftsman plot showing pairwise scatterplots between eMDI features; each point represents an individual sample.
