# Supplementary material for: The Submucosal Microbiome Correlates with Peri-implantitis Severity
Source: J Dent Res. 2025 Jul 28;105(3):313–22. doi: 10.1177/00220345251352809 (PMC12861548; doi:10.1177/00220345251352809)
Supplement: sj-docx-2-jdr-10.1177_00220345251352809 – Supplemental material for The Submucosal Microbiome Correlates with Peri-implantitis Severity [file sj-docx-2-jdr-10.1177_00220345251352809.docx]

STROBE Statement—Checklist of items that should be included in reports of ***cross-sectional studies***

|  | Item No | Recommendation | Refer in manuscript |
| --- | --- | --- | --- |
| **Title and abstract** | 1 | (*a*) Indicate the study’s design with a commonly used term in the title or the abstract | Page 2 line 27 |
|  |  | (*b*) Provide in the abstract an informative and balanced summary of what was done and what was found | Page 2 lines 27-44 |
| Introduction | | |  |
| Background/rationale | 2 | Explain the scientific background and rationale for the investigation being reported | Page 3 lines 46-60 |
| Objectives | 3 | State specific objectives, including any prespecified hypotheses | Page 4 lines 76-81 |
| Methods | | |  |
| Study design | 4 | Present key elements of study design early in the paper | Page 4 lines 83-86 |
| Setting | 5 | Describe the setting, locations, and relevant dates, including periods of recruitment, exposure, follow-up, and data collection | Page 4 lines 86-93; Appendix page 2 lines 26-34 |
| Participants | 6 | (*a*) Give the eligibility criteria, and the sources and methods of selection of participants | Appendix page 16 Table 1 |
| Variables | 7 | Clearly define all outcomes, exposures, predictors, potential confounders, and effect modifiers. Give diagnostic criteria, if applicable | Page 5 lines 96-100; Appendix page 2 lines 26-33 |
| Data sources/ measurement | 8* | For each variable of interest, give sources of data and details of methods of assessment (measurement). Describe comparability of assessment methods if there is more than one group | Appendix methods page 2-3 lines 42-51 |
| Bias | 9 | Describe any efforts to address potential sources of bias | Appendix methods Appendix page 8 lines 184-194 |
| Study size | 10 | Explain how the study size was arrived at | Appendix page 3 lines 51-60 |
| Quantitative variables | 11 | Explain how quantitative variables were handled in the analyses. If applicable, describe which groupings were chosen and why | Page 5 lines 110-117 |
| Statistical methods | 12 | (*a*) Describe all statistical methods, including those used to control for confounding | Appendix page 7-9 lines 163-216 |
|  |  | (*b*) Describe any methods used to examine subgroups and interactions | n/a. No subgroups or interactions were specifically examined in the study. |
|  |  | (*c*) Explain how missing data were addressed | n/a. No missing data was present for the variable of interest in the study. |
|  |  | (*d*) If applicable, describe analytical methods taking account of sampling strategy | n/a. The selection of patients and implants was based on clinical criteria rather than a predefined sampling strategy. |
|  |  | (*e*) Describe any sensitivity analyses | n/a. The primary analysis focused on examining the main associations between variables. |
| Results | | |  |
| Participants | 13* | (a) Report numbers of individuals at each stage of study—eg numbers potentially eligible, examined for eligibility, confirmed eligible, included in the study, completing follow-up, and analysed | Page 5 lines 96-98 |
|  |  | (b) Give reasons for non-participation at each stage | n/a. All eligible individuals participated in the study. |
|  |  | (c) Consider use of a flow diagram | n/a |
| Descriptive data | 14* | (a) Give characteristics of study participants (eg demographic, clinical, social) and information on exposures and potential confounders | Appendix page 17 - Table 2 |
|  |  | (b) Indicate number of participants with missing data for each variable of interest | n/a. No missing data was present for the variable of interest in the study. |
| Outcome data | 15* | Report numbers of outcome events or summary measures | Page 5 lines 103-105; page 7 lines 146-148; page 8 lines 168-170 |
| Main results | 16 | (*a*) Give unadjusted estimates and, if applicable, confounder-adjusted estimates and their precision (eg, 95% confidence interval). Make clear which confounders were adjusted for and why they were included | Page 6 lines 128-130; Page 7 lines 149-151; Appendix page 7 lines 173-194 |
|  |  | (*b*) Report category boundaries when continuous variables were categorized | Page 5 lines 114-117 |
|  |  | (*c*) If relevant, consider translating estimates of relative risk into absolute risk for a meaningful time period | n/a. The emphasis is on the correlation and prediction strength rather than absolute risk measures |
| Other analyses | 17 | Report other analyses done—eg analyses of subgroups and interactions, and sensitivity analyses | n/a |
| Discussion | | |  |
| Key results | 18 | Summarise key results with reference to study objectives | Page 9 lines 195-200 |
| Limitations | 19 | Discuss limitations of the study, taking into account sources of potential bias or imprecision. Discuss both direction and magnitude of any potential bias | Page 12-13 lines 291-308 |
| Interpretation | 20 | Give a cautious overall interpretation of results considering objectives, limitations, multiplicity of analyses, results from similar studies, and other relevant evidence | Page 10-12 lines 219-290 |
| Generalisability | 21 | Discuss the generalisability (external validity) of the study results | Page 10 lines 233-235; page 12 lines 291-293 |
| Other information | | |  |
| Funding | 22 | Give the source of funding and the role of the funders for the present study and, if applicable, for the original study on which the present article is based | Page 14 lines 334-336 |

*Give information separately for exposed and unexposed groups.

**Note:** An Explanation and Elaboration article discusses each checklist item and gives methodological background and published examples of transparent reporting. The STROBE checklist is best used in conjunction with this article (freely available on the Web sites of PLoS Medicine at http://www.plosmedicine.org/, Annals of Internal Medicine at http://www.annals.org/, and Epidemiology at http://www.epidem.com/). Information on the STROBE Initiative is available at www.strobe-statement.org.
